# Temporal change in characteristics and outcomes of acute kidney injury on renal replacement therapy in intensive care units: analysis of a nationwide administrative database in Japan, 2007–2016

**DOI:** 10.1186/s13054-019-2468-8

**Published:** 2019-05-15

**Authors:** Yoshihisa Miyamoto, Masao Iwagami, Shotaro Aso, Hideo Yasunaga, Hiroki Matsui, Kiyohide Fushimi, Yoshifumi Hamasaki, Masaomi Nangaku, Kent Doi

**Affiliations:** 1 Division of Nephrology and Endocrinology, The University of Tokyo, 7-3-1 Hongo, Bunkyo-ku, Tokyo, 113-0033 Japan; 2 Department of Health Services Research, University of Tsukuba, 1-1-1 Tennodai, Tsukuba, Ibaraki 305-8575 Japan; 3 Department of Clinical Epidemiology and Health Economics, School of Public Health, The University of Tokyo, 7-3-1 Hongo, Bunkyo-ku, Tokyo, 113-0033 Japan; 4 Department of Health Policy and Informatics, Tokyo Medical and Dental University Graduate School of Medicine, Tokyo, Japan; 5 Department of Hemodialysis and Apheresis, The University of Tokyo Hospital, 7-3-1 Hongo, Bunkyo-ku, Tokyo, 113-0033 Japan; 6 Department of Acute Care Medicine, The University of Tokyo Hospital, 7-3-1 Hongo, Bunkyo-ku, Tokyo, 113-0033 Japan

**Keywords:** Acute kidney injury, Cardiovascular disease, Epidemiology, Renal replacement therapy, Sepsis

## Abstract

**Background:**

We aimed to examine recent trends in patient characteristics and mortality in patients with acute kidney injury (AKI) receiving renal replacement therapy (RRT), including continuous RRT (CRRT) and intermittent RRT (IRRT), in intensive care units (ICUs).

**Methods:**

From the Diagnosis Procedure Combination database in Japan during 6 months (July–December) from 2007 to 2016, we identified patients with AKI who received RRT in ICUs. We restricted the study participants to those admitted to hospitals (in which both CRRT and IRRT were available) that participated in the Diagnosis Procedure Combination database for all 10 years. We examined the trends in patient characteristics and mortality overall, by RRT modality, and by main diagnosis category subgroup. Logistic regression was used to adjust for patient characteristics.

**Results:**

We identified 51,758 patients starting RRT in 287 hospitals, including 39,471 (76.3%) and 12,287 (23.7%) patients starting CRRT and IRRT. The crude in-hospital mortality declined from 44.9 to 36.1% (*P* for trend < 0.001). Compared with 2007, the adjusted odds ratio (aOR) for in-hospital mortality was 0.66 (95% confidence interval (CI) 0.60–0.72) in 2016, and the decreasing trend was observed in both patients starting CRRT (aOR 0.67, 95% CI 0.61–0.75) and IRRT (0.58, 0.45–0.74), and in all subgroups except for coronary artery disease: sepsis aOR 0.68 (95% CI 0.57–0.81); cardiovascular surgery 0.58 (0.45–0.76); coronary artery disease 0.84 (0.60–1.19); non-coronary heart disease 0.78 (0.64–0.94); central nervous system disorders 0.42 (0.28–0.62); trauma 0.39 (0.21–0.72); and other 0.64 (0.50–0.82).

**Conclusions:**

This nationwide study confirmed a consistent decline in mortality among patients with AKI on RRT in ICUs. The adjusted mortality also declined during the study period; however, physiological variables were not measured in this study and it is possible that RRT may have been indicated for patients with less severe AKI in more recent years.

**Electronic supplementary material:**

The online version of this article (10.1186/s13054-019-2468-8) contains supplementary material, which is available to authorized users.

## Background

Acute kidney injury (AKI) is common in critically ill patients and is strongly associated with high mortality [1]. Renal replacement therapy (RRT) is an important supportive therapy for patients with AKI. In particular, continuous renal replacement therapy (CRRT) is used for hemodynamically unstable critically ill patients with loss of kidney function [2]. Previous studies showed that the overall mortality of patients with AKI requiring CRRT was high—over 50% [2, 3]—indicating that further improvement was necessary for this unacceptable poor outcome.

There have been a number of reports on a recent decrease in mortality among critically ill patients [4–10]. One study showed a 35% relative decrease in mortality among patients admitted to intensive care units (ICUs) in the USA from 1988 to 2012, despite an increase in age and severity of disease [4]. Among patients with AKI on dialysis (AKI-D), another study in the USA showed that in-hospital mortality consistently decreased from 1992 to 2002 [10]. In contrast, one study from the UK reported that the unadjusted mortality of patients with AKI-D was around 30% from 1998 to 2007 but gradually increased to over 40% in 2013 [7]. This increasing trend in in-hospital mortality was significant even after adjusting for changes in patient characteristics over time.

Previously, we reported the characteristics and outcomes of patients with AKI-D in Japan at 2011 using a nationwide claims database, suggesting poor outcomes of AKI-D in ICUs [3, 11]. Comprehension of temporal trends in baseline characteristics including organ supportive therapies with RRT, RRT modalities, and outcomes in patients with AKI-D is highly relevant to physicians. Therefore, using the same nationwide inpatient database, we investigated the recent trends in characteristics and in-hospital mortality among patients treated with RRT for AKI in ICUs in Japan over a decade, from 2007 to 2016. Furthermore, we examined whether the temporal change in in-hospital mortality differed by RRT modality [CRRT or intermittent RRT (IRRT)] and by the main diagnosis of patients on RRT: sepsis, cardiovascular surgery, coronary artery disease, non-coronary heart disease, central nervous system (CNS) disorders, trauma, or other.

## Methods

### Data source

For the present study, we used the Diagnosis Procedure Combination (DPC) inpatient database. The details of the database have been described elsewhere [3]. Briefly, the DPC database is a national administrative claim and discharge abstract database in Japan. This database contains data of approximately half of all inpatient admissions to acute care hospitals and covers over 90% of all tertiary care emergency hospitals in Japan. Data available in the database include patient’s age and sex; admission and discharge dates; discharge status (deceased or living); ICU admissions (including emergency rooms, coronary care units, and stroke care units); primary diagnosis at admission, comorbidities at admission, and post-admission complications coded using International Classification of Diseases, 10th revision (ICD-10) codes; types of surgery coded with the original Japanese operation codes; and procedures (including CRRT and IRRT) and drugs used during hospitalization on a daily basis. All clinically relevant data were recorded by attending physicians at the time of hospital discharge. A validation study of the DPC database has suggested high sensitivity and specificity of procedure records, while most diagnoses had high specificity but moderate sensitivity [12].

Study approval was obtained from the institutional review board of The University of Tokyo. Because data were anonymized, the requirement to obtain informed consent from individual patients was waived.

### Study participants

In the DPC database, data were collected for 6 months (July through December) from 2007 to 2010 and throughout the year from 2011 to 2016. To improve the comparability of data across calendar years, we compared data of patients who were discharged in the same season from July to December over the 10-year period between 2007 and 2016. In addition, we restricted the study participants to those admitted to hospitals (in which both CRRT and IRRT were available) that participated in the DPC database for 10 consecutive years.

The study participants were therefore patients aged 18 years or older starting RRT (CRRT or IRRT) in ICUs of hospitals that contributed to the DPC database for the 10 study years, in every included 6-month period (July–December) from 2007 to 2016. We excluded patients with the end-stage renal disease based on the ICD-10 code of N18.0 at admission and/or evidence of maintenance hemodialysis.

### Definition of variables and study outcomes

We categorized the eligible patients into seven main diagnosis categories based on ICD-10 and operation codes and procedures: (i) sepsis group, based on the ICD-10 codes suggesting infection (e.g., pneumonia, cholecystitis, or pyelonephritis) [13] and evidence of other organ dysfunction (mechanical ventilation, vasoactive drugs, or platelet transfusion) at the initiation of RRT [14]; (ii) cardiovascular surgery group, based on the diagnosis category of “disease of the circulatory system” (ICD-10 codes I00–I99) and Japanese operation codes indicating cardiovascular surgery; (iii) coronary artery disease group, based on the diagnosis category of heart disease (ICD-10 codes I00–I52) and Japanese operation codes indicating percutaneous coronary intervention (PCI); (iv) non-coronary heart disease group, based on the diagnosis category of heart disease (ICD-10 codes I00–I52) without cardiovascular surgery or PCI; (v) CNS disorders group, based on the ICD-10 codes suggesting CNS disorders (ICD-10 codes C70–C72, G03–G09, G35–37, G40–41, and I60–I69); (vi) trauma group, based on the ICD-10 codes for injuries (S00–T14); and (vii) other. In categorizing patients with multiple conditions, the primary diagnosis was assigned the highest priority, followed by comorbidity at admission and then post-admission complication, so that a patient was classified into one of the seven subgroups.

We identified the route of ICU admission: emergency room, operation room, or ward. We calculated the Charlson comorbidity index (CCI) score based on the information on comorbidities at admission [15] and the duration (days) from hospital admission to RRT initiation. We identified patients with a recorded diagnosis of chronic kidney disease (CKD) as a complication at admission (ICD-10 code; N18.X except for N18.0). The diagnostic code for CKD can be used to stratify patients, but may be limited with regard to its low sensitivity in administrative databases [16]. Further, we identified the following treatments performed on the day of RRT initiation: mechanical ventilation; transfusion including red blood cell concentrates, fresh frozen plasma, or platelets; vasoactive agents including dopamine, dobutamine, norepinephrine, or epinephrine; intra-aortic balloon pumping; extracorporeal membrane oxygenation; and plasma exchange.

The primary outcome of interest was overall in-hospital mortality. The secondary outcomes were the length of hospital stay and dialysis dependence among hospital survivors. Dialysis dependence was defined as the use of RRT within 2 days of discharge (i.e., on the day of discharge or 1 day prior to the discharge date) among hospital survivors.

### Statistical analysis

We first examined whether patient characteristics changed from 2007 to 2016, using the Jonckheere–Terpstra trend test for age as a continuous variable and the Cochrane–Armitage test for binary and categorical variables. We then examined whether the crude in-hospital mortality of patients on RRT changed over the 10-year study period, using the Cochrane–Armitage test. We determined if the temporal change in outcomes in the AKI-D population was specific or was in line with a general trend in ICUs over the 10-year study period by comparing this population with patients without AKI-D in the same ICUs over the same 10 years and tested the trend using the Cochrane–Armitage test.

We then conducted multivariable-adjusted generalized estimating equation logistic regression analysis to examine the association between year and in-hospital mortality, clustering by hospitals (to account for inter-hospital correlation) and adjusting for baseline patient characteristics (age, sex, main diagnosis category, route of ICU admission, CCI score, recorded diagnosis of CKD, duration from hospital admission to RRT initiation, RRT modality, and treatments on the day of RRT initiation). In the subgroup analyses, we examined the 10-year trend in crude in-hospital mortality and adjusted odds ratio (aOR) for in-hospital mortality separately by RRT modality (i.e., CRRT and IRRT) and by main diagnosis category (i.e., sepsis, cardiovascular surgery, coronary artery disease, non-coronary heart disease, CNS disorders, trauma, and other). We also performed subgroup analyses stratified by age group (below 65, 65–74, 75–84, over 85 years), sex, CKD status, and mechanical ventilation status at RRT initiation.

As a post hoc analysis, we demonstrated trends in the proportion of co-interventions separately by subgroup to elucidate potential mechanisms of difference in mortality trend between subgroups.

A two-sided *P* <  0.05 was considered statistically significant. Data were analyzed using SPSS version 25 and R software version 3.5.0.

## Results

### Temporal changes in patient characteristics

We identified 51,758 adult patients starting RRT for AKI, including 39,471 (76.3%) patients starting CRRT and 12,287 (23.7%) patients starting IRRT, in 287 hospitals (consisting of 59 [20.6%] academic and 228 [79.4%] non-academic hospitals) that participated in the DPC database continuously for 10 years from 2007 to 2016. The proportion of patients with AKI-D in ICUs generally decreased from 4.3% in 2007 to 3.7% in 2016, with some fluctuations (Table 1). The proportion of patients starting CRRT among patients with AKI-D slightly decreased from 77.8% in 2007 to 74.5% in 2016 (*P* for trend < 0.001).

**Table 1:** Characteristics of patients treated with renal replacement therapy for acute kidney injury in intensive care units from 2007 to 2016

	Year										*P* for trend
	2007	2008	2009	2010	2011	2012	2013	2014	2015	2016	
Number of patients on RRT	3892	4139	3526	4917	5255	5788	5841	6065	6365	5970	
Proportion of AKI-D patients in all ICU patients (%)	4.3	4.6	3.6	4.1	4.0	3.8	3.7	3.6	3.7	3.7	
Age (years, mean)	67.5	67.7	68.3	68.0	68.1	68.5	68.3	68.8	69.0	68.7	< 0.001
Sex (male, %)	65.3	66.5	65.8	65.9	65.7	65.9	65.1	66.9	65.7	64.7	0.481
Main diagnosis category (%)											
(i) Sepsis	23.3	25.4	26.4	27.5	26.6	25.8	26.6	26.0	26.3	27.5	0.002
(ii) Cardiovascular surgery	13.3	13.4	15.0	14.9	14.8	15.3	14.9	15.6	15.8	14.8	< 0.001
(iii) Coronary artery disease	10.8	10.1	9.8	7.8	7.9	7.1	6.9	7.0	7.1	6.9	< 0.001
(iv) Non-coronary heart disease	27.0	27.4	25.9	26.1	26.6	27.8	26.9	26.8	27.3	27.1	0.451
(v) Central nervous system disorders	6.0	6.5	6.1	6.2	6.3	6.1	6.2	5.9	6.3	6.5	0.736
(vi) Trauma	2.1	2.2	2.0	2.7	3.0	3.3	3.0	3.4	2.7	3.3	< 0.001
(vii) Other	17.5	15.1	14.7	14.8	14.7	14.6	15.6	15.3	14.6	13.9	0.002
Route of ICU admission (%)											
Emergency room	61.4	61.5	61.5	59.0	59.2	59.6	61.3	63.3	62.5	62.6	< 0.001
Operation room	16.5	17.9	18.9	22.3	23.8	24.5	23.7	22.7	24.7	24.8	< 0.001
Ward	22.1	20.6	19.6	18.8	17.0	15.9	15.0	14.0	12.9	12.5	< 0.001
Charlson comorbidity index (%)											
Score 0	33.1	30.8	29.8	28.8	29.0	28.9	29.3	28.5	29.0	27.4	< 0.001
Score 1	16.5	16.9	16.9	15.7	16.6	16.4	16.2	16.5	15.3	14.7	< 0.001
Score 2	25.4	26.4	25.8	26.2	26.6	25.3	25.1	26.6	26.2	25.7	< 0.001
Score 3	14.1	13.9	14.4	15.2	14.8	15.7	15.8	14.8	15.9	15.8	< 0.001
Score ≥ 4	10.9	12.1	13.2	14.0	13.1	13.7	13.5	13.6	13.6	16.3	< 0.001
Chronic kidney disease (%)	24.6	26.3	25.0	25.4	26.5	26.9	26.7	26.7	26.4	26.6	0.01
Duration from hospital admission to RRT initiation (days, median [IQR])	3 [1, 13]	3 [2, 12]	3 [2, 13]	3 [2, 13]	3 [2, 13]	3 [2, 12]	3 [2, 11]	3 [2, 10]	3 [2, 9]	3 [2, 9]	< 0.001
RRT modality (CRRT, %)	77.8	80.5	81.7	79.8	77.1	75.1	74.5	73.5	73.1	74.5	< 0.001
Treatment on day of RRT initiation (%)											
Mechanical ventilation	49.8	50.8	52.1	52.8	51.1	51.1	50.0	49.6	49.8	50.9	0.114
Any blood transfusion	45.4	46.0	45.7	50.9	50.4	49.0	48.3	47.7	46.3	47.6	0.486
Red blood cell concentrates	37.5	38.5	37.9	43.0	43.2	41.8	40.8	40.4	39.5	40.8	0.026
Fresh frozen plasma	25.6	25.3	25.9	28.5	28.0	27.9	28.3	27.4	25.8	26.3	0.465
Platelets	16.1	17.0	15.7	18.9	18.8	19.2	18.1	18.2	17.1	16.6	0.500
Any vasoactive drug	62.6	61.9	62.8	61.8	61.2	59.0	57.6	57.7	56.7	58.1	< 0.001
Dopamine	53.2	50.7	51.0	49.6	45.9	40.5	35.2	30.8	26.8	24.4	< 0.001
Dobutamine	24.0	24.0	23.5	21.2	20.5	21.3	20.6	20.1	20.5	20.6	< 0.001
Norepinephrine	31.7	32.5	33.3	35.8	38.4	38.8	41.0	43.4	44.0	46.1	< 0.001
Epinephrine	12.1	11.5	11.3	10.8	10.8	9.8	10.5	10.3	10.6	10.6	0.003
Intra-aortic balloon pumping	6.8	7.4	7.4	6.5	6.5	6.7	6.8	7.0	6.8	6.2	0.162
Extracorporeal membrane oxygenation	2.1	2.3	2.4	2.5	2.7	2.7	3.1	3.1	2.9	3.3	< 0.001
Plasma exchange	2.6	2.4	2.6	3.2	2.7	2.2	2.6	2.3	2.4	1.9	0.008

*CKD* chronic kidney disease, *CRRT* continuous renal replacement therapy, *ICU* intensive care unit, *IQR* interquartile range, *RRT* renal replacement therapy

Patient characteristics are shown by year in Table 1. The mean age of ICU patients on RRT increased from 67.5 years in 2007 to 68.7 years in 2016 (*P* for trend < 0.001). The proportions of men and women did not change significantly over time (*P* for trend = 0.481), with the men making up approximately 65% of the included patients throughout the study period. The distribution of main diagnosis category changed significantly over time. Notably, the percentage with a main diagnosis of sepsis as the main diagnosis category increased from 23.3% in 2007 to 27.5% in 2016. There was an increasing trend in the CCI score over the 10-year study period.

The median duration from hospital admission to RRT initiation was constantly 3 days throughout the study period. On the day of RRT initiation, there was no significant change in the percentage of mechanical ventilation (at approximately 50%) and blood transfusion (at approximately 45%). The percentage of vasoactive agents significantly decreased from 62.6% in 2007 to 58.1% in 2016, whereas its details changed largely. The percentage of patients on dopamine decreased from 53.2% in 2007 to 24.4% in 2016, whereas the percentage receiving norepinephrine increased from 31.7% to 46.1% over the same time period. Patient characteristics by RRT modality (CRRT and IRRT) are shown in Additional file 1.

### Temporal changes in outcomes

**Fig. 1:**
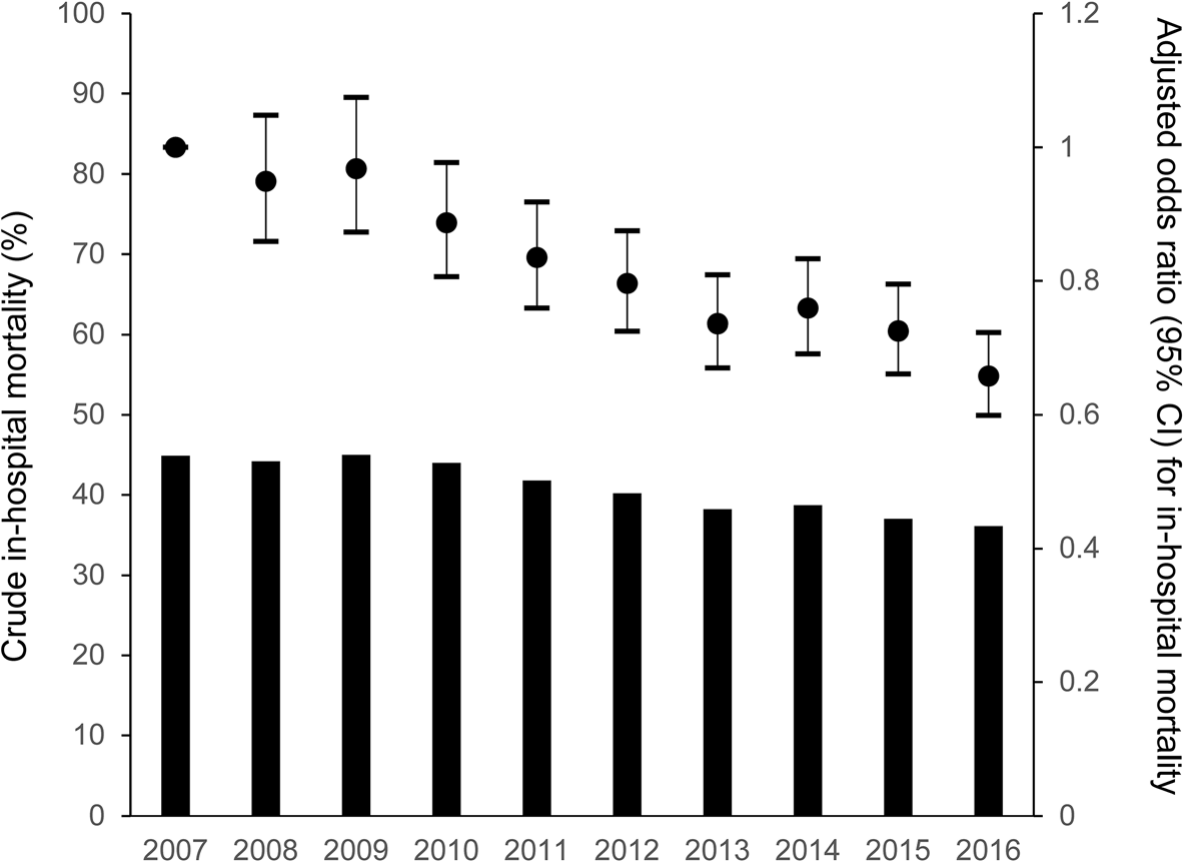
Temporal trend in crude in-hospital mortality and adjusted odds ratios for in-hospital mortality among patients treated with renal replacement therapy in intensive care units from 2007 to 2016. Note: The left axis denotes crude in-hospital mortality, and the right axis denotes the adjusted odds ratio for in-hospital mortality. The error bars indicate 95% confidence intervals (CIs) for the adjusted odds ratios

The overall crude in-hospital mortality of ICU patients with AKI-D consistently decreased from 44.9% in 2007 to 36.1% in 2016 (*P* for trend < 0.001, Fig. 1). After adjusting for baseline characteristics, the decreasing trend of in-hospital mortality was prominent (Fig. 1 and Table 2): Compared with patients receiving RRT in 2007, the aOR for in-hospital mortality was 0.66 (95% confidence interval (CI) 0.60–0.79) among patients receiving RRT in 2016 (Table 2).

**Table 2:** Outcomes of patients treated with renal replacement therapy for acute kidney injury in intensive care units from 2007 to 2016

	Year										*P* for trend
	2007	2008	2009	2010	2011	2012	2013	2014	2015	2016	
In-hospital mortality, %	44.9	44.2	45.0	44.0	41.8	40.2	38.2	38.7	37.0	36.1	< 0.001
Adjusted odds ratio (95% CI) for in-hospital mortality*	1 (ref)	0.95 (0.86, 1.06)	0.97 (0.87, 1.08)	0.89 (0.80, 0.99)	0.84 (0.75, 0.94)	0.80 (0.72, 0.89)	0.74 (0.66, 0.83)	0.76 (0.68, 0.85)	0.72 (0.65, 0.81)	0.66 (0.59, 0.74)	n/a
Length of hospital stay (median [IQR]) among hospital survivors	39 [21, 68]	37 [19, 66]	36 [21, 64]	40 [23, 68]	42 [23, 77]	41 [23, 75]	40 [21, 72]	38 [21, 69]	37 [21, 68]	36 [20, 65]	0.660
Proportion of dialysis dependence (%) among hospital survivors	15.7	17.2	15.6	16.9	18.2	19.3	19.0	19.3	19.0	19.4	< 0.001

*CI* confidence interval, *IQR* interquartile range, *n/a* not available

*Adjusted for age, sex, main diagnosis category, route of intensive care unit admission, Charlson comorbidity index score, coded with CKD, duration from hospital admission to renal replacement therapy initiation, renal replacement therapy modality, and treatment on the day of renal replacement therapy initiation (mechanical ventilation, any blood transfusion, any vasoactive drug, intra-aortic balloon pumping, extracorporeal membrane oxygenation, and plasma exchange)

There was no significant change in the length of hospital stay among hospital survivors (*P* for trend = 0.660), with median 35–40 days over the 10-year study period (Table 2). The proportion of dialysis dependence among hospital survivors increased from 15.7% in 2007 to 19.4% in 2016 (*P* for trend < 0.001).

**Fig. 2:**
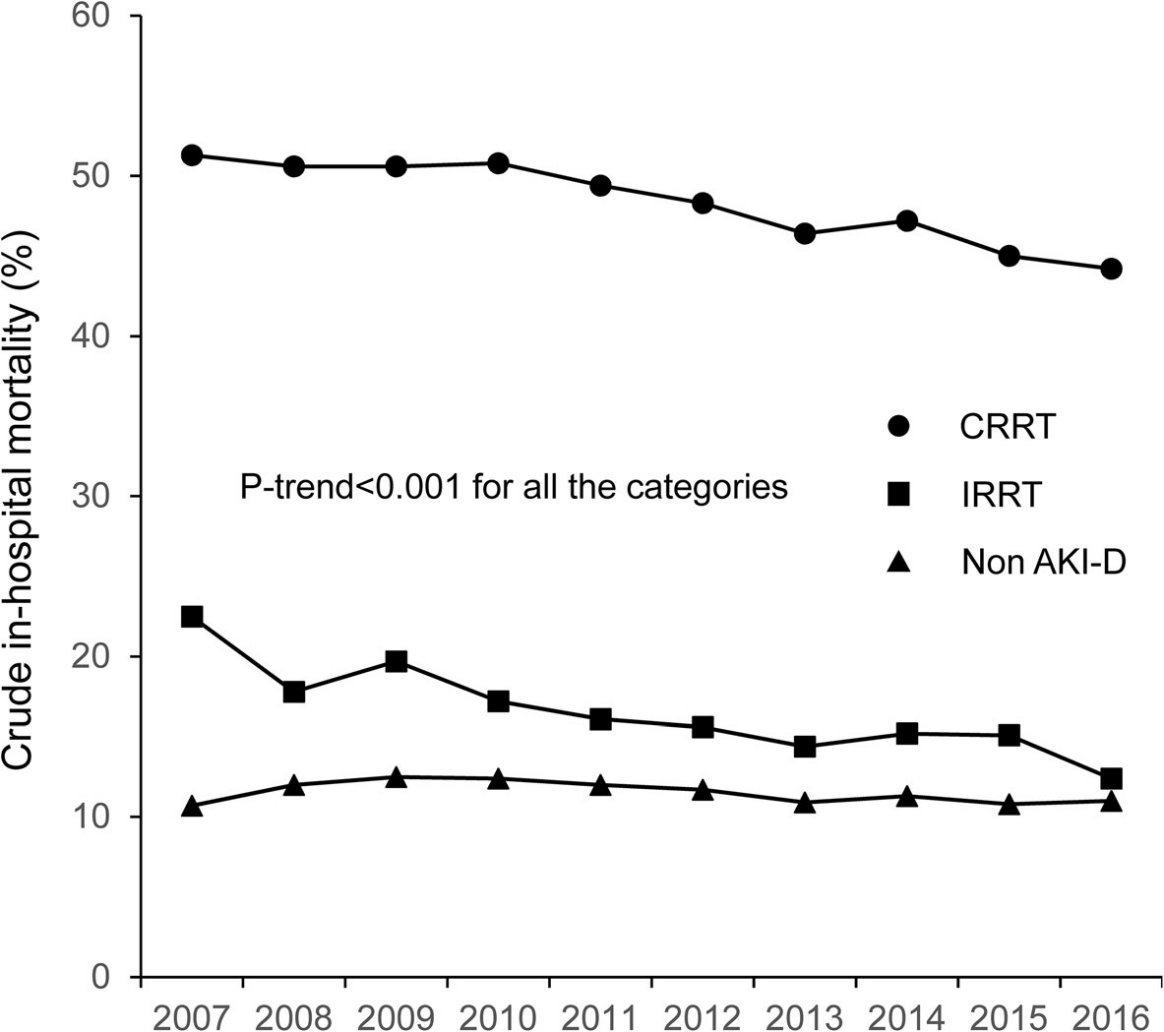
Temporal changes in crude in-hospital mortality among patients with continuous and intermittent renal replacement therapy and patients without dialysis for acute kidney injury in intensive care units from 2007 to 2016. Abbreviations: AKI-D acute kidney injury with dialysis, CRRT continuous renal replacement therapy, IRRT intermittent renal replacement therapy

In the subgroup analysis by RRT modality, the crude in-hospital mortality of patients starting CRRT constantly decreased from 51.3% in 2007 to 44.2% in 2016 (*P* for trend < 0.001), whereas that of patients starting IRRT constantly decreased from 22.5% in 2007 to 12.4% in 2016 (*P* for trend < 0.001) (Fig. 2). Figure 2 also demonstrates the in-hospital mortality of patients without AKI-D in the same ICUs. There was a statistically significant decreasing trend (*P* for trend < 0.001), but the in-hospital mortality did not appear to be clinically very different over the 10-year study period, at approximately 11–12%.

**Fig. 3:**
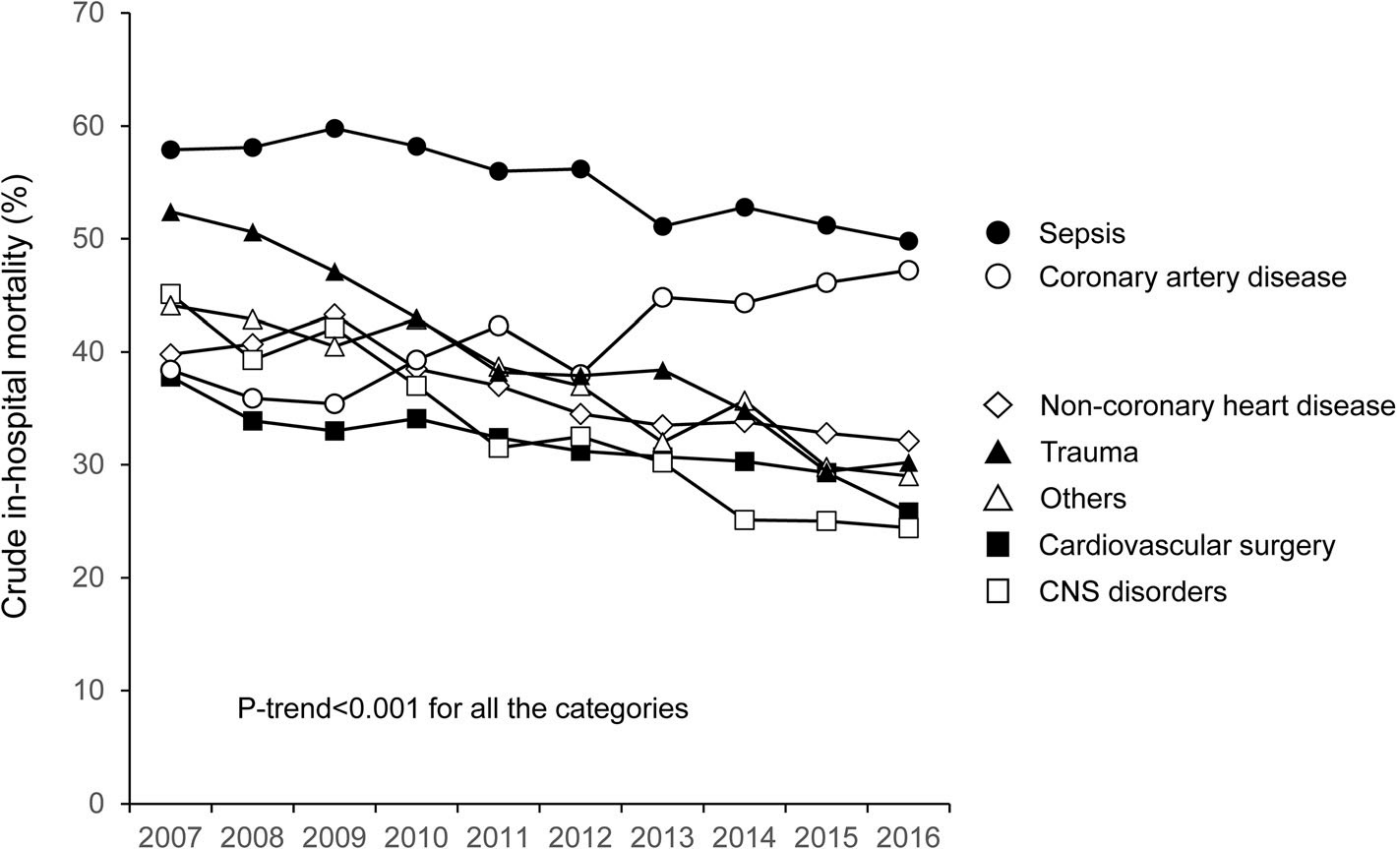
Temporal change in crude in-hospital mortality by main diagnosis category among patients treated with renal replacement therapy in intensive care units from 2007 to 2016. Abbreviation: CNS central nervous system

In the subgroup analysis by main diagnosis category (Fig. 3), the crude in-hospital mortality decreased in all subgroups except for the coronary artery disease group (from 38.4% in 2007 to 47.2% in 2016). In the adjusted analyses (Table 3), all groups except for coronary artery disease showed improved in-hospital mortality in 2016 compared with 2007: the aOR (95% CI) was as follows: (i) sepsis, 0.68 (0.57–0.81); (ii) cardiovascular surgery, 0.58 (0.45–0.76); (iii) coronary artery disease, 0.84 (0.60–1.19); (iv) non-coronary heart disease, 0.78 (0.64–0.94); (v) CNS disorders, 0.42 (0.28–0.62); (vi) trauma, 0.39 (0.21–0.72); and (vii) other, 0.64 (0.50–0.82).

**Table 3:** Subgroup analyses: adjusted odds ratio for in-hospital mortality by renal replacement therapy modality and by main diagnosis category

	Year									
	2007	2008	2009	2010	2011	2012	2013	2014	2015	2016
Subgroup by RRT modality^a^										
(i) Patients starting CRRT	1 (ref.)	0.99 (0.89,1.10)	0.97 (0.87,1.09)	0.90 (0.81,1.00)	0.85 (0.77,0.95)	0.80 (0.73,0.89)	0.74 (0.67,0.82)	0.76 (0.69,0.85)	0.73 (0.66,0.80)	0.67 (0.61,0.75)
(ii) Patients starting IRRT	1 (ref.)	0.74 (0.56,0.97)	0.96 (0.23,1.27)	0.79 (0.61,1.02)	0.73 (0.57,0.93)	0.75 (0.59,0.95)	0.70 (0.55,0.89)	0.72 (0.57,0.91)	0.70 (0.55,0.89)	0.58 (0.45,0.74)
Subgroup by main diagnosis category^b^										
(i) Sepsis	1 (ref.)	1.00 (0.82,1.20)	1.09 (0.89,1.32)	0.93 (0.78,1.12)	0.87 (0.73,1.04)	0.85 (0.71,1.02)	0.69 (0.58,0.83)	0.77 (0.65,0.92)	0.74 (0.62,0.87)	0.68 (0.57,0.81)
(ii) Cardiovascular surgery	1 (ref.)	0.82 (0.63,1.09)	0.85 (0.64,1.12)	0.90 (0.69,1.17)	0.82 (0.64,1.07)	0.77 (0.59,0.99)	0.79 (0.61,1.02)	0.74 (0.58,0.96)	0.73 (0.57,0.93)	0.58 (0.45,0.76)
(iii) Coronary artery disease	1 (ref.)	0.95 (0.67,1.35)	0.82 (0.57,1.19)	0.81 (0.57,1.15)	0.92 (0.65,1.30)	0.71 (0.50,1.00)	0.73 (0.52,1.03)	0.85 (0.60,1.19)	0.83 (0.59,1.16)	0.84 (0.60,1.19)
(iv) Non-coronary heart disease	1 (ref.)	1.11 (0.91,1.35)	1.14 (0.93,1.40)	0.93 (0.77,1.13)	0.90 (0.75,1.10)	0.87 (0.72,1.05)	0.83 (0.69,1.00)	0.84 (0.69,1.01)	0.86 (0.71,1.03)	0.78 (0.64,0.94)
(v) CNS disorders	1 (ref.)	0.76 (0.50,1.13)	0.98 (0.64,1.50)	0.64 (0.43,0.96)	0.52 (0.35,0.77)	0.54 (0.36,0.79)	0.58 (0.40,0.86)	0.41 (0.28,0.61)	0.45 (0.31,0.67)	0.42 (0.28,0.62)
(vi) Trauma	1 (ref.)	0.72 (0.36,1.46)	0.62 (0.30,1.29)	0.73 (0.39,1.39)	0.52 (0.28,0.97)	0.58 (0.32,1.07)	0.61 (0.33,1.12)	0.52 (0.29,0.95)	0.41 (0.22,0.76)	0.39 (0.21,0.72)
(vii) Other	1 (ref.)	0.97 (0.74,1.25)	0.85 (0.65,1.12)	0.98 (0.76,1.26)	0.90 (0.76,1.26)	0.88 (0.69,1.13)	0.76 (0.59,0.97)	0.89 (0.70,1.14)	0.70 (0.55,0.90)	0.64 (0.50,0.82)
Subgroup by age (years)^c^										
(i) < 65	1 (ref.)	1.17 (0.90, 1.28)	1.04 (0.86, 1.25)	0.91 (0.77, 1.08)	0.85 (0.72, 1.00)	0.82 (0.70, 0.98)	0.79 (0.67, 0.94)	0.79 (0.67, 0.94)	0.83 (0.70, 0.98)	0.68 (0.57, 0.80)
(ii) 65–74	1 (ref.)	0.90 (0.75, 1.09)	1.05 (0.86, 1.27)	0.94 (0.79, 1.12)	0.87 (0.73, 1.04)	0.86 (0.72, 1.02)	0.66 (0.55, 0.78)	0.78 (0.66, 0.93)	0.70 (0.59, 0.83)	0.68 (0.57, 0.81)
(iii) 74–85	1 (ref.)	0.89 (0.74, 1.06)	0.86 (0.71, 1.04)	0.84 (0.70, 1.00)	0.74 (0.63, 0.88)	0.75 (0.64, 0.89)	0.73 (0.62, 0.86)	0.70 (0.59, 0.82)	0.63 (0.53, 0.74)	0.61 (0.52, 0.72)
(iv) > 85	1 (ref.)	1.01 (0.70, 1.48)	0.89 (0.62, 1.28)	0.79 (0.55, 1.12)	1.02 (0.72, 1.44)	0.70 (0.50, 0.98)	0.79 (0.56, 1.11)	0.80 (0.57, 1.10)	0.76 (0.55, 1.05)	0.63 (0.45, 0.87)
Subgroup by sex^d^										
(i) Male	1 (ref.)	0.95 (0.84, 1.08)	0.99 (0.87, 1.13)	0.89 (0.79, 1.00)	0.86 (0.76, 0.97)	0.81 (0.72, 0.91)	0.76 (0.67, 0.85)	0.76 (0.68, 0.86)	0.73 (0.65, 0.82)	0.66 (0.59, 0.75)
(ii) Female	1 (ref.)	0.96 (0.82, 1.14)	0.94 (0.79, 1.12)	0.90 (0.77, 1.06)	0.80 (0.69, 0.94)	0.77 (0.66, 0.89)	0.70 (0.60, 0.82)	0.74 (0.64, 0.87)	0.71 (0.61, 0.83)	0.65 (0.55, 0.76)
Subgroup by CKD^e^										
(i) With CKD	1 (ref.)	0.91 (0.73, 1.13)	0.96 (0.76, 1.21)	0.75 (0.60, 0.93)	0.76 (0.61, 0.94)	0.77 (0.62, 0.94)	0.73 (0.59, 0.90)	0.71 (0.57, 0.87)	0.70 (0.57, 0.86)	0.63 (0.51, 0.78)
(ii) Without CKD	1 (ref.)	0.97 (0.87, 1.09)	0.97 (0.87, 1.10)	0.93 (0.84, 1.04)	0.86 (0.77, 0.96)	0.81 (0.73, 0.90)	0.74 (0.67, 0.83)	0.77 (0.70, 0.86)	0.74 (0.66, 0.82)	0.67 (0.60, 0.74)
Subgroup by mechanical ventilation^f^										
(i) Receiving mechanical ventilation at RRT start	1 (ref.)	0.92 (0.81, 1.06)	0.93 (0.81, 1.07)	0.91 (0.80, 1.03)	0.79 (0.69, 0.89)	0.75 (0.66, 0.85)	0.66 (0.58, 0.75)	0.72 (0.64, 0.82)	0.69 (0.61, 0.78)	0.65 (0.57, 0.74)
(ii) Not receiving mechanical ventilation at RRT start	1 (ref.)	0.99 (0.85, 1.15)	1.01 (0.86, 1.18)	0.84 (0.72, 0.97)	0.89 (0.76, 1.02)	0.85 (0.74, 0.98)	0.84 (0.73, 0.97)	0.80 (0.70, 0.92)	0.75 (0.65, 0.86)	0.65 (0.56, 0.75)

*CKD* chronic kidney disease, *CI* confidence interval, *CNS* central nervous system, *CRRT* continuous renal replacement therapy, *IRRT* intermittent renal replacement therapy, *RRT* renal replacement therapy

^a^Adjusted for the covariates used in the main analysis (see Table 2) except for RRT modality

^b^Adjusted for the covariates used in the main analysis (see Table 2) except for the main diagnosis category

^c^Adjusted for covariates used in the main analysis (see Table 2) except for age category

^d^Adjusted for covariates used in the main analysis (see Table 2) except for sex

^e^Adjusted for covariates used in the main analysis (see Table 2) except for CKD

^f^Adjusted for covariates used in the main analysis (see Table 2) except for mechanical ventilation

Subgroup analyses by age group, sex, CKD, and mechanical ventilation showed similar trends in both crude mortality and aOR compared with the general trend over the 10 years (Additional file 1: File 4, Table 3). We also observed increasing trends in RRT dependence at discharge in patients both with and without CKD over the 10-year period (Additional file 1: File 5).

In post hoc analysis, there was an increasing trend of percentages of patients receiving vasoactive agents, blood transfusion, mechanical ventilation, intra-aortic balloon pumping, and extracorporeal membrane oxygenation in the coronary artery disease group (*P* for trend < 0.001 in all of these covariates; Additional file 1), whereas no such trends were observed in other subgroups.

## Discussion

In this nationwide study conducted in Japan, we found that the crude in-hospital mortality of ICU patients treated with RRT for AKI consistently decreased from 44.9% in 2007 to 36.1% in 2016, although several characteristics of these patients (e.g., age, CCI score, main diagnosis category, and details of vasoactive drugs) were changing over time. Moreover, the adjusted analysis supported the decreasing trend in the in-hospital mortality of patients with AKI-D in ICUs. The aOR of in-hospital mortality improved in both patients starting CRRT and IRRT and in all subgroups except for coronary artery disease according to the main diagnosis category.

Although the absolute number of patients with AKI-D increased, the proportion of patients with AKI-D in ICUs in the 287 studied hospitals generally decreased from 4.3% in 2007 to 3.7% in 2016, reflecting an increase in the number of patients admitted to ICUs over the 10-year period. This suggests that acute RRT was not necessarily becoming more common in ICUs in terms of proportion, but the actual burden of care for AKI-D was apparently increasing in real-world clinical practice, in terms of the absolute number of patients with AKI-D and those dying of AKI-D.

We observed that the mean age of patients treated with RRT increased over the 10-year study period. This may reflect the recent increasing trend in the proportion of elderly patients in ICUs, which has been suggested by previous studies [7, 17, 18]. In a Danish cohort, the percentage of patients aged over 65 years who were admitted to ICUs increased from 11.7% in 2005 to 13.8% in 2011 [17]. The incidence rate of AKI-D among people aged over 65 years doubled from 2000 to 2009 in the USA [18], and the mean age of patients with AKI-D increased from 59.7 years in 1998–1999 to 65.1 years in 2012–2013 in England [7]. Our finding of an increase in mean patient age from 67.6 years in 2007 to 68.9 years in 2016 was consistent with these studies. Moreover, we found an increasing trend in the CCI score among patients with AKI-D in line with the UK nationwide inpatient study [7], although this may be partly explained by the potential improvement of diagnosis recording over time.

We observed a substantial decline in the unadjusted and adjusted in-hospital mortality of patients on RRT in accordance with previous nationwide studies regarding AKI-D [4, 9, 19]. We observed a downward trend in in-hospital mortality in both patients starting CRRT and IRRT and in each group of patients categorized according to the main diagnosis category. Potential explanation of these findings may include general improvements in the management of critical care (e.g., sepsis management according to the Surviving Sepsis Campaign Guideline). However, this hypothesis does not appear to be consistent with the temporal trend in the mortality of ICU patients without AKI-D, which did not change substantially during the study period (Fig. 2). We do not consider that the choice of RRT modality was the main contributor of the decreasing mortality, because the proportion of CRRT (vs. IRRT) changed only slightly during the study period (from 77.8% in 2007 to 74.5% in 2016) and the downward trend of mortality was observed regardless of the RRT modality.

It is also possible that the decrease in mortality of patients with AKI-D was related to the more liberal use of RRT (i.e., starting RRT in a wider group of patients with less-severe AKI) and/or the selective use of RRT (i.e., excluding patients whose condition was considered to be too severe, for whom RRT was considered futile). Although the proportion of RRT patients with co-interventions (such as mechanical ventilation and blood transfusion) did not change significantly over the study period, these patients may have become less sick in terms of physiological variables, which were not measured in this present study. Although several RCTs have validated the effect of RRT timing on prognosis in patients with AKI [20–22], controversies remain over the optimal timing and/or target patients for starting RRT. The current results (i.e., decreasing trend in in-hospital mortality among patients with AKI-D) may reflect temporal changes in clinicians’ behavior regarding RRT indication, and this possibility should be explored in future studies.

While in-hospital mortality was declined over 10 years, the proportion of dialysis dependence among hospital survivors increased from 15.7% in 2007 to 19.4% in 2016. This suggests that, as a consequence of the improvement in in-hospital mortality of patients with AKI-D, the burden of non-recovery from AKI-D has been increasing in the community. A recent study from the USA showed that patients with non-recovery from AKI-D are at high risk of cardiovascular events [19]. According to our study results, more attention may be needed to cope with the increasing burden of non-recovery from AKI-D. We observed decreasing trends in mortality and increasing trends in RRT-dependence at discharge in patients both with and without CKD over a 10-year period. The higher mortality of AKI-D patients without CKD compared with those with CKD was in line with previous studies, suggesting that patients with advanced CKD required a less severe insult to initiate RRT [23, 24], given that they are generally more prone to AKI than patients without CKD [25].

Despite the general decreasing trend in crude in-hospital mortality, we observed an increasing trend of the crude in-hospital mortality in coronary artery disease group (from 38.4% in 2007 to 47.2% in 2016). In additional analysis, we observed that the percentages of patients with vasoactive agents, blood transfusion, mechanical ventilation, intra-aortic balloon pumping, and extracorporeal membrane oxygenation increased in the coronary artery disease group, whereas these trends were not observed in other subgroups (Additional file 1: File 3). Notably, after adjusting for patient characteristics including co-interventions, the in-hospital mortality did not significantly change from 2007 to 2016 (aOR 0.85, 95% CI 0.60–1.20), meaning that the increase in the crude in-hospital mortality can be explained by an increase in the severity of illness in AKI-D patients with coronary artery disease over the 10 years. The increase in severity of illness may be potentially due to temporal changes in indication for PCI, which needs to be explored in future studies.

The present study has several limitations. First, our findings may not be generalizable to the whole country, because the DPC inpatient database is not based on a random sample of Japanese hospitals. Furthermore, we restricted the study participants to those admitted to hospitals that participated in the DPC database continuously from 2007 to 2016 to maintain comparability across different calendar years. Considering that most tertiary care hospitals and academic hospitals have been participating in the DPC database, participants in the current study were probably more severe than patients admitted to other hospitals in Japan. Second, we classified patients by main diagnosis category based using ICD-10 codes, in which misclassification would be concerned. However, we also used operation and procedure codes, which are generally known to have good validity in the database [12]. Third, the low sensitivity of the diagnostic coding for AKI [26] meant that we were unable to assess the trend in mortality for patients with AKI without RRT. It therefore remains unknown if the decreasing mortality was the result of improved care for AKI or improved management of acute RRT. Further studies including patients with AKI without RRT are warranted to resolve this issue. Fourth, we were unable to obtain data on the details of RRT, such as blood flow rates and dialysate/filtration fluid flow rates. Therefore, we cannot deny the possibility that the decreasing mortality of patients on RRT may be explained in part by changes in RRT practice over time, although the prognostic impact of RRT intensity is still controversial [27]. Fifth, in the present study, which used only data on the DPC inpatient database, data were available only during hospital admission. We acknowledge that mortality at a fixed time point (e.g., 30-day, 60-day, and 90-day mortality) would be a more suitable outcome than in-hospital mortality, which could be influenced by a change in discharge policy over time. However, we demonstrated that the length of hospital stay among hospital survivors did not significantly change during the study period (Table 2), meaning that the decreasing in-hospital mortality of patients with AKI-D cannot be explained by early discharge policy. Finally, although we adjusted for a wide range of covariates obtained in the database, there may be unmeasured confounders, such as vital signs, volume status, laboratory data including baseline creatinine, and inotropic score, which potentially reflect changes in the indication for RRT over the 10 years.

## Conclusions

This nationwide study in Japan suggested a consistent decrease in the in-hospital mortality among patients treated with RRT for AKI from 2007 to 2016, although several patient characteristics were changing over time. The adjusted mortality also declined during the study period; however, it is possible that RRT was indicated for patients with less-severe AKI in more recent years. Further studies including measurements of physiological variables are therefore required to explain the exact mechanisms responsible for the decrease in mortality in this important patient group in ICUs.

## Additional file

Additional file 1: File 1 Characteristics of patients treated with continuous renal replacement therapy for acute kidney injury in intensive care units from 2007 to 2016. File 2 Characteristics of patients treated with intermittent renal replacement therapy for acute kidney injury in intensive care units from 2007 to 2016. File 3 Trends in the proportion of co-interventions by subgroups in patients treated with renal replacement therapy for acute kidney injury in intensive care units from 2007 to 2016. File 4 Subgroup analyses: in-hospital mortality of patients treated with renal replacement therapy for acute kidney injury in intensive care units from 2007 to 2016. (PDF 2665 kb)

## Abbreviations

AKI: Acute kidney injury
AKI-D: Acute kidney injury requiring dialysis
CCI: Charlson comorbidity index
CI: Confidence interval
CKD: Chronic kidney disease
CNS: Central nervous system
CRRT: Continuous renal replacement therapy
DPC: Diagnosis Procedure Combination
ICD: The International Classification of Diseases
IRRT: Intermittent renal replacement therapy
OR: Odds ratio
PCI: Percutaneous coronary intervention
RRT: Renal replacement therapy
