# Supplementary material for: Temporal change in characteristics and outcomes of acute kidney injury on renal replacement therapy in intensive care units: analysis of a nationwide administrative database in Japan, 2007–2016
Source: Crit Care. 2019 May 15;23:172. doi: 10.1186/s13054-019-2468-8 (PMC6521368; doi:10.1186/s13054-019-2468-8)
Supplement: Supplementary file 1 — File 1 Characteristics of patients treated with continuous renal replacement therapy for acute kidney injury in intensive care units from 2007 to 2016. File 2 Characteristics of patients treated with intermittent renal replacement therapy for acute kidney injury in intensive care units from 2007 to 2016. File 3 Trends in the proportion of co-interventions by subgroups in patients treated with renal replacement therapy for acute kidney injury in intensive care units from 2007 to 2016. File 4 Subgroup analyses: in-hospital mortality of patients treated with renal replacement therapy for acute kidney injury in intensive care units from 2007 to 2016. (PDF 2665 kb) [file 13054_2019_2468_MOESM1_ESM.pdf]

Online Supplementary Material

Temporal change in characteristics and outcomes of acute kidney injury on renal replacement therapy in intensive care units: Analysis of a nationwide administrative database in Japan, 2007–2016

Yoshihisa Miyamoto, Masao Iwagami, Shotaro Aso, Hideo Yasunaga, Hiroki Matsui, Kiyohide Fushimi, Yoshifumi Hamasaki, Masaomi Nangaku, Kent Doi

Contents:

File 1. Characteristics of patients treated with continuous renal replacement therapy for acute kidney injury in intensive care units from 2007 to 2016

File 2. Characteristics of patients treated with intermittent renal replacement therapy for acute kidney injury in intensive care units from 2007 to 2016

File 3. Trends in the proportion of co-interventions by subgroups in patients treated with renal replacement therapy for acute kidney injury in intensive care units from 2007 to 2016

File 4. Subgroup analyses: in-hospital mortality of patients treated with renal replacement therapy for acute kidney injury in intensive care units from 2007 to 2016

File 5. The proportion of RRT dependence at discharge stratified by CKD, in patients treated with renal replacement therapy for acute kidney injury in intensive care units from 2007 to 2016

File 1. Characteristics of patients treated with continuous renal replacement therapy for acute kidney injury in intensive care units from 2007 to 2016

Year	2007	2008	2009	2010	2011	2012	2013	2014	2015	2016	P for trend
Number of patients on CRRT	3027	3330	2882	3922	4050	4348	4352	4458	4655	4447	
Age (years, mean)	67.6	68.0	68.5	68.0	68.3	68.7	68.5	69.0	68.9	68.9	<0.001
Sex (male, %)	64.2	65.9	65.5	66.0	65.2	66.1	65.1	66.8	65.6	64.1	0.955
Main diagnosis category (%)											
i) sepsis	27.3	29.3	30.4	32.6	32.0	32.3	33.1	32.8	33.4	34.7	<0.001
ii) cardiovascular surgery	14.6	14.5	16.1	16.1	16.1	17.2	16.4	16.9	17.6	16.1	<0.001
iii) coronary artery disease	10.1	9.5	9.3	7.7	8.5	7.8	8.0	7.8	8.2	8.4	<0.001
iv) non-coronary heart disease	25.0	25.2	24.2	23.2	23.8	23.7	23.2	23.3	22.7	22.6	<0.001
v) central nervous system disorders	5.1	4.9	4.2	4.8	4.7	4.7	4.2	4.2	4.6	4.5	0.150
vi) trauma	1.9	2.1	2.0	2.4	2.5	2.5	2.5	2.6	1.9	2.7	0.120
vii) other	16.0	14.5	13.7	13.2	12.4	11.9	12.7	12.4	11.7	11.1	<0.001
Route of ICU admission (%)											
Emergency room	58.1	59.5	60.0	58.7	59.5	60.7	62.6	64.7	65.0	64.9	<0.001
Operation room	16.3	17.3	18.2	19.8	20.5	20.5	19.5	18.8	19.7	20.2	<0.001
Ward	25.5	23.2	21.8	21.5	20.0	18.7	17.9	16.4	15.3	14.9	<0.001
Charlson comorbidity index (%)											
Score 0	35.1	32.9	31.7	31.4	31.8	32.4	32.3	31.8	32.3	30.2	0.003
Score 1	14.6	14.2	14.9	13.0	13.3	12.6	12.4	12.9	11.9	11.5	<0.001
Score 2	26.0	27.7	27.1	27.8	28.9	27.5	27.2	29.0	28.6	28.3	0.020
Score 3	13.0	13.1	12.9	13.8	13.4	14.2	14.4	12.8	14.2	14.1	0.056
Score ≥4	11.4	12.1	13.5	14.0	12.7	13.4	13.7	13.5	13.0	15.9	<0.001
Chronic kidney disease (%)	20.5	21.7	21.1	20.4	20.0	20.1	19.3	19.0	18.5	19.3	<0.001
Duration from hospital admission to CRRT initiation (days, median [IQR])	4 [2, 14]	3 [2, 13]	4 [2, 14]	4 [2, 15]	4 [2, 15]	4 [2, 15]	4 [2, 14]	4 [2, 13]	3 [2, 12]	3 [2, 11]	<0.001

(*Continued*)

File 1. (CONTINUED)

Treatment on day of CRRT initiation (%)											
Mechanical ventilation	57.7	58.5	59.5	62.3	61.6	63.0	62.1	62.5	62.6	63.4	<0.001
Any blood transfusion	53.0	52.2	51.9	59.3	59.3	59.2	59.1	58.6	56.6	58.2	<0.001
(Red blood cell concentrates)	43.5	43.9	43.0	50.0	50.7	50.6	50.3	49.5	48.4	50.0	<0.001
(Fresh frozen plasma)	30.7	29.7	30.1	34.4	34.5	35.5	36.0	35.6	33.4	33.7	<0.001
(Platelets)	19.4	19.9	18.4	22.8	23.3	24.5	22.9	23.4	22.2	21.4	<0.001
Any vasoactive drug	73.4	71.4	72.7	73.3	74.6	73.9	73.1	73.7	72.8	74.0	0.211
(Dopamine)	62.8	58.4	59.0	59.3	56.4	51.2	45.2	40.1	35.0	31.2	<0.001
(Dobutamine)	28.5	28.1	27.6	25.1	25.1	27.1	26.4	26.1	26.8	26.6	0.081
(Norepinephrine)	38.5	39.4	39.6	43.4	47.6	49.6	52.9	56.5	57.5	59.6	<0.001
(Epinephrine)	15.0	13.8	13.6	13.2	13.6	12.8	13.9	13.8	14.2	14.0	0.993
Intra-aortic balloon pumping	8.0	8.8	8.8	8.0	8.0	8.8	9.0	9.3	9.1	8.3	0.184
Extracorporeal membrane oxygenation	2.7	2.8	2.9	3.1	3.5	3.6	4.1	4.2	3.9	4.4	<0.001
Plasma exchange	2.8	2.3	2.4	3.5	2.9	2.5	2.8	2.6	2.5	1.9	0.030

CRRT = continuous renal replacement therapy, ICU = intensive care unit, IQR = interquartile range.

File 2. Characteristics of patients treated with intermittent renal replacement therapy for acute kidney injury in intensive care units from 2007 to 2016

Year	2007	2008	2009	2010	2011	2012	2013	2014	2015	2016	P for trend
Number of patients on IRRT	865	809	644	995	1205	1440	1489	1607	1710	1523	
Age (years, mean)	67.2	66.7	67.2	68.0	67.4	67.7	67.9	68.1	69.3	68.3	<0.001
Sex (male, %)	68.9	69.1	67.2	65.4	67.6	65.6	65.2	67.4	65.8	66.3	0.098
Main diagnosis category (%)											
i) sepsis	9.0	9.1	8.4	7.6	8.4	6.2	7.5	7.0	7.1	6.4	0.002
ii) cardiovascular surgery	9.0	8.8	10.1	10.2	10.7	9.5	10.5	11.9	11.2	11.0	0.010
iii) coronary artery disease	13.3	12.5	12.3	8.0	6.0	5.1	3.8	4.9	3.9	2.7	<0.001
iv) non-coronary heart disease	33.8	36.7	33.5	37.6	36.0	40.1	37.8	36.5	39.7	40.4	<0.001
v) central nervous system disorders	9.5	12.9	14.8	11.5	11.5	10.4	12.0	10.7	10.9	12.3	0.871
vi) trauma	2.7	2.5	1.7	4.0	4.7	5.8	4.6	5.6	4.7	5.3	<0.001
vii) other	22.8	17.6	19.3	21.1	22.7	22.9	23.8	23.3	22.5	21.9	0.043
Route of ICU admission (%)											
Emergency room	72.7	69.7	68.0	59.9	58.3	56.0	57.4	59.4	55.6	56.1	<0.001
Operation room	17.2	20.4	22.0	32.1	34.9	36.5	36.2	33.4	38.0	38.3	<0.001
Ward	10.1	9.9	9.9	8.0	6.9	7.5	6.4	7.2	6.4	5.6	<0.001
Charlson comorbidity index (%)											
Score 0	26.1	22.0	21.3	18.6	19.4	18.5	20.8	19.1	20.1	19.2	0.002
Score 1	23.4	27.8	25.8	26.6	27.7	27.7	27.3	26.6	24.5	24.2	0.355
Score 2	23.1	20.6	19.9	19.7	18.8	18.8	19.1	20.1	19.8	18.1	0.032
Score 3	18.0	17.6	21.3	20.8	19.8	20.5	19.9	20.2	20.2	20.9	0.105
Score ≥4	9.4	12.0	11.8	14.3	14.3	14.5	12.9	13.9	15.4	17.6	<0.001
Chronic kidney disease (%)	38.8	45.2	42.7	45.0	48.1	47.4	48.3	37.9	47.8	48.1	<0.001
Duration from hospital admission to RRT initiation (days, median [IQR])	2 [1, 9]	2 [1, 7]	2 [1, 9]	2 [1, 6]	2 [2, 4]	2 [1, 4]	2 [1, 4]	2 [1, 4]	2 [1, 4]	2 [1, 4]	<0.001

(*Continued*)

File 2. (CONTINUED)

Treatment on day of IRRT initiation (%)											
Mechanical ventilation	22.3	19.0	19.3	15.3	15.8	15.0	14.6	13.8	15.1	14.4	<0.001
Any blood transfusion	19.0	20.1	18.2	17.9	20.6	18.3	16.7	17.7	18.1	16.3	0.021
(Red blood cell concentrates)	16.5	16.1	15.2	15.5	18.0	15.5	13.2	15.2	15.1	14.0	0.033
(Fresh frozen plasma)	7.5	7.3	6.8	5.0	6.1	4.9	5.9	4.6	5.0	4.7	<0.001
(Platelets)	4.3	4.9	3.6	3.2	3.4	3.0	4.1	3.7	3.1	2.6	0.013
Any vasoactive drug	24.7	23.0	18.2	16.5	16.3	14.0	12.2	13.1	12.9	11.8	<0.001
(Dopamine)	19.8	19.0	15.2	11.1	10.5	8.3	5.9	5.0	4.8	4.5	<0.001
(Dobutamine)	8.3	7.3	5.0	5.8	5.0	3.6	3.5	3.6	3.5	3.2	<0.001
(Norepinephrine)	8.0	4.3	5.3	5.5	7.6	6.2	6.2	7.2	7.1	6.4	0.271
(Epinephrine)	2.2	2.3	1.2	1.1	1.1	0.8	0.6	0.7	0.8	0.8	<0.001
Intra-aortic balloon pumping	2.5	1.7	0.8	1.0	1.3	0.5	0.5	0.7	0.6	0.3	<0.001
Extracorporeal membrane oxygenation	0.1	0.0	0.0	0.1	0.1	0.2	0.2	0.1	0.2	0.0	NA
Plasma exchange	1.8	2.7	3.3	1.8	1.7	1.3	1.8	1.5	2.1	2.0	0.303

ICU = intensive care unit, IQR = interquartile range, IRRT = intermittent renal replacement therapy.

File 3: Trends in the proportion of co-interventions by subgroups in patients treated with renal replacement therapy for acute kidney injury in intensive care units from 2007 to 2016

CNS = central nervous system, ECMO = extracorporeal membrane oxygenation, IABP= intra-aortic balloon pumping, NA=not available, RRT = renal replacement therapy

P-values for trend were not available for co-interventions identifying no cases in some years.

(To the Next Page)

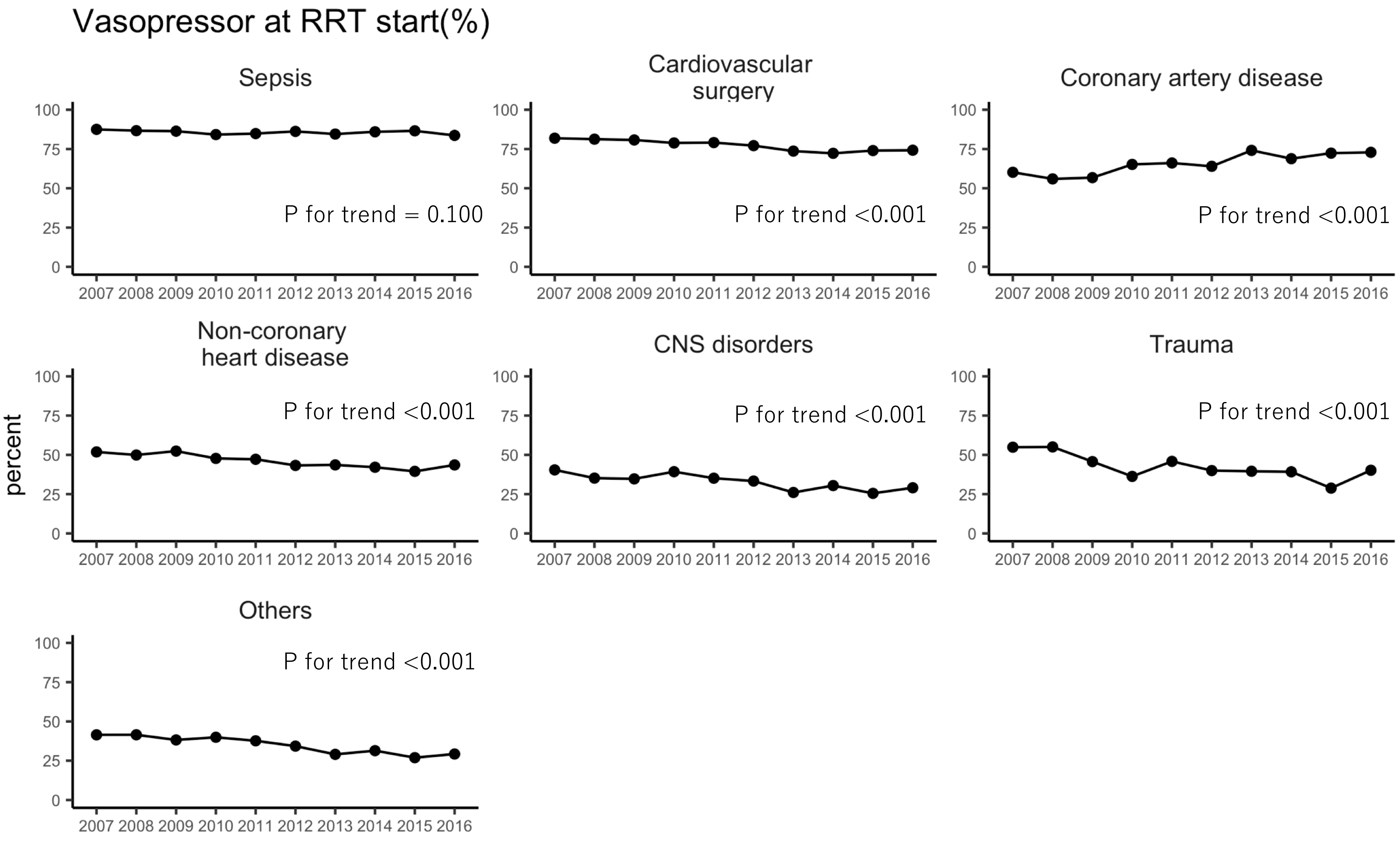
Vasopressor at RRT start(%)
Sepsis
P for trend = 0.100
Cardiovascular surgery
P for trend <0.001
Coronary artery disease
P for trend <0.001
Non-coronary heart disease
P for trend <0.001
CNS disorders
P for trend <0.001
Trauma
P for trend <0.001
Others
P for trend <0.001
percent
0
25
50
75
100
2007 2008 2009 2010 2011 2012 2013 2014 2015 2016

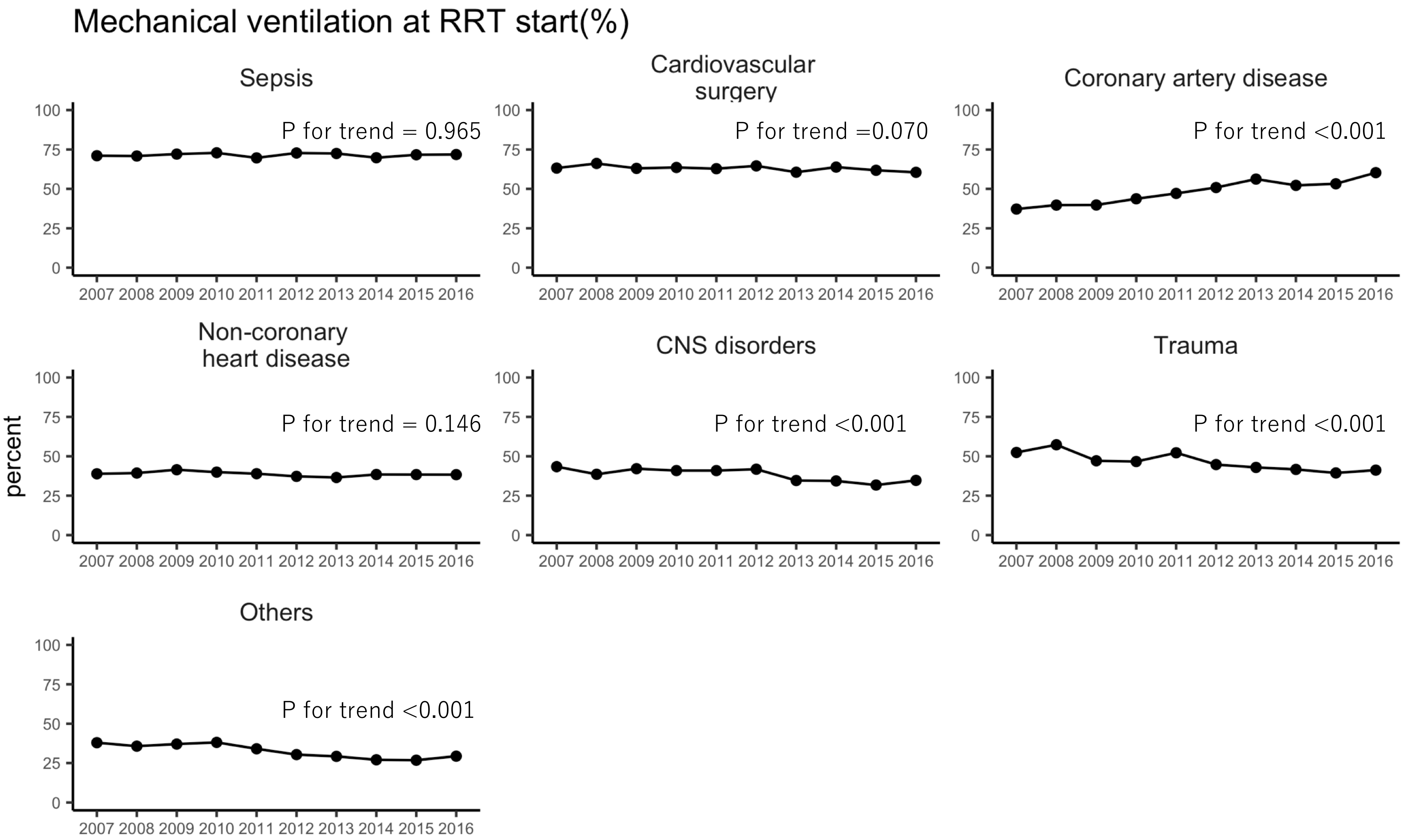

Mechanical ventilation at RRT start(%)
Sepsis
P for trend = 0.965
Cardiovascular surgery
P for trend =0.070
Coronary artery disease
P for trend <0.001
Non-coronary heart disease
P for trend = 0.146
CNS disorders
P for trend <0.001
Trauma
P for trend <0.001
Others
P for trend <0.001
percent
0
25
50
75
100
2007 2008 2009 2010 2011 2012 2013 2014 2015 2016

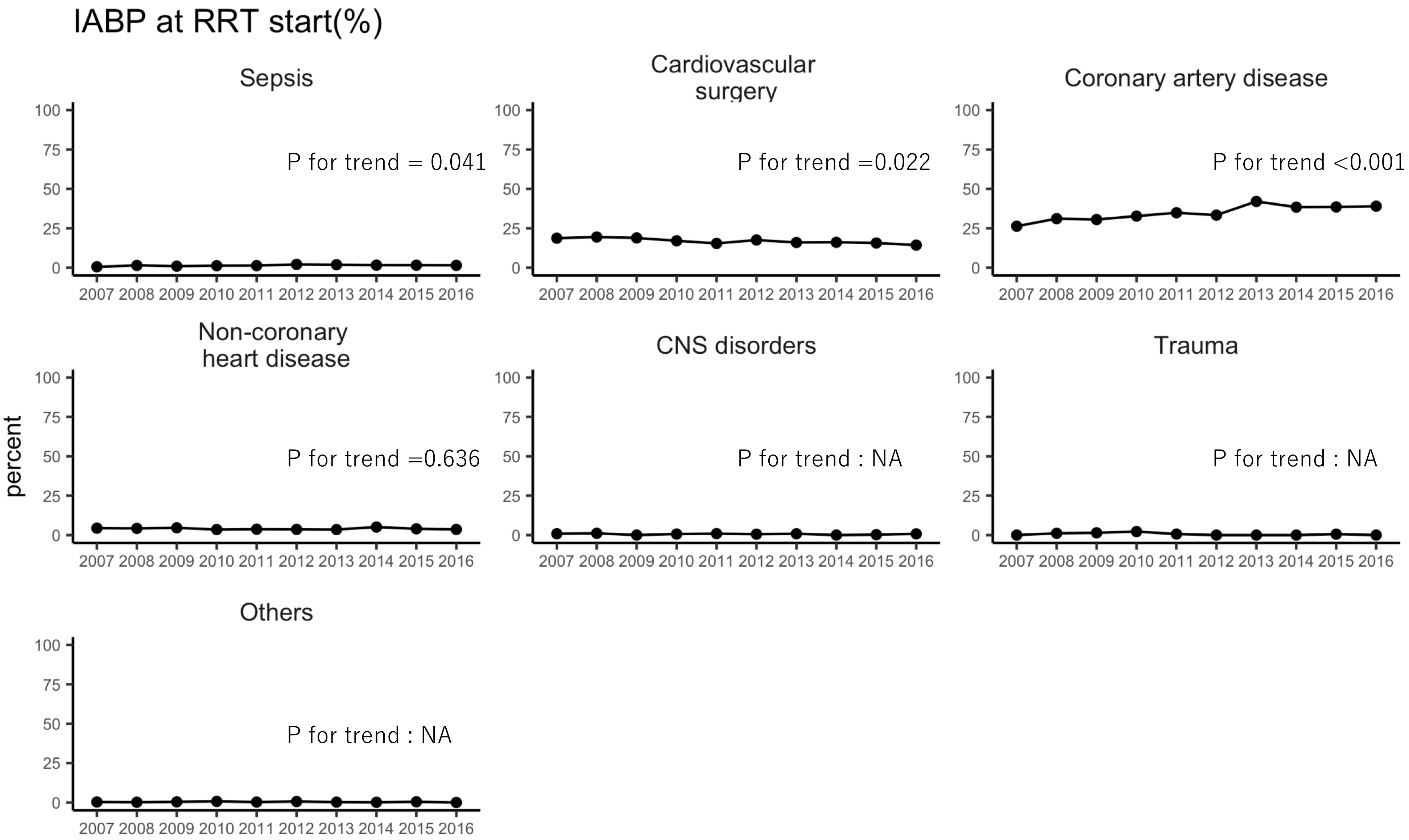

IABP at RRT start(%)
Sepsis
P for trend = 0.041
Cardiovascular surgery
P for trend =0.022
Coronary artery disease
P for trend <0.001
Non-coronary heart disease
P for trend =0.636
CNS disorders
P for trend : NA
Trauma
P for trend : NA
Others
P for trend : NA
percent
0 25 50 75 100
2007 2008 2009 2010 2011 2012 2013 2014 2015 2016

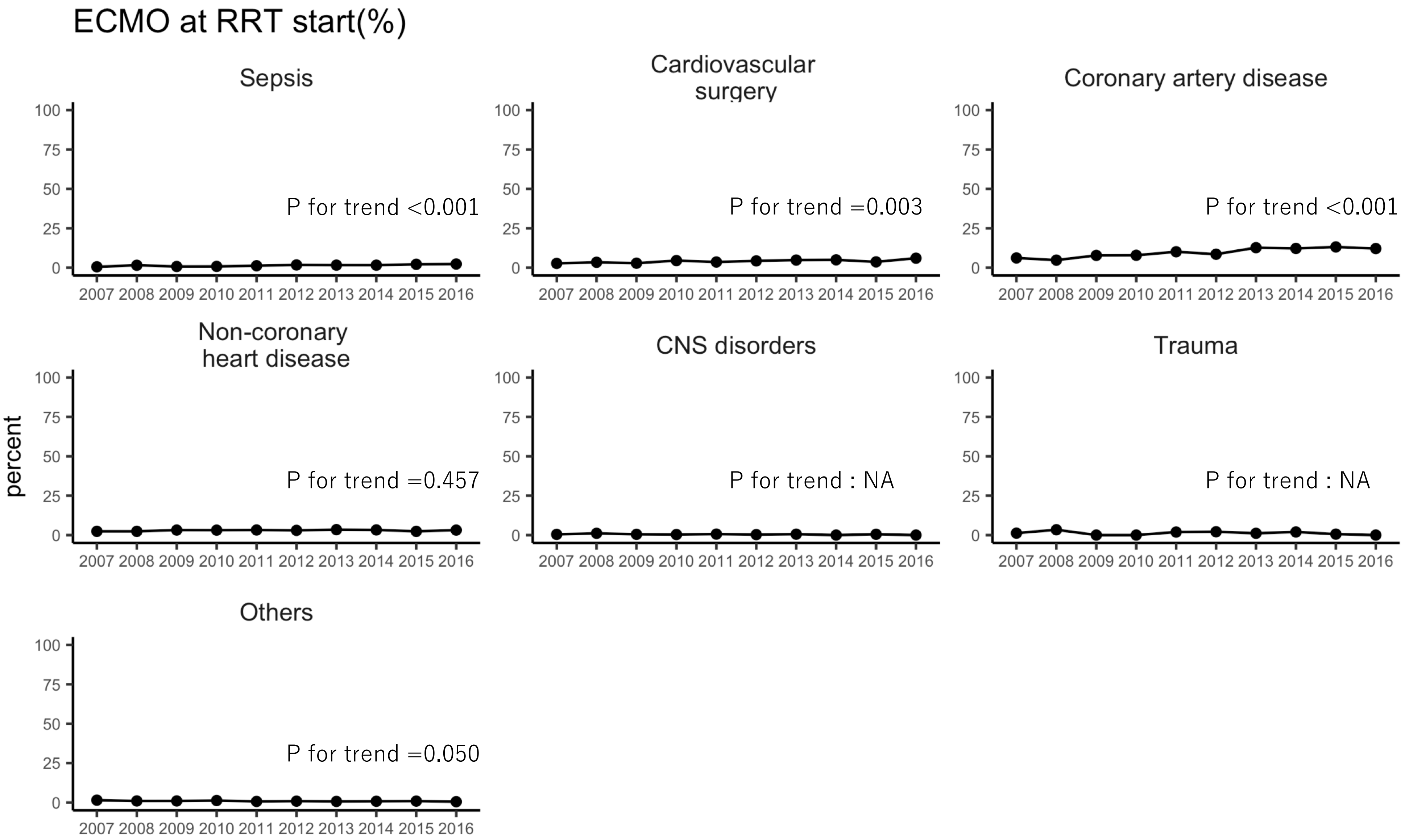
ECMO at RRT start(%)
percent
Sepsis
P for trend <0.001
Cardiovascular surgery
P for trend =0.003
Coronary artery disease
P for trend <0.001
Non-coronary heart disease
P for trend =0.457
CNS disorders
P for trend : NA
Trauma
P for trend : NA
Others
P for trend =0.050
0
25
50
75
100
2007 2008 2009 2010 2011 2012 2013 2014 2015 2016

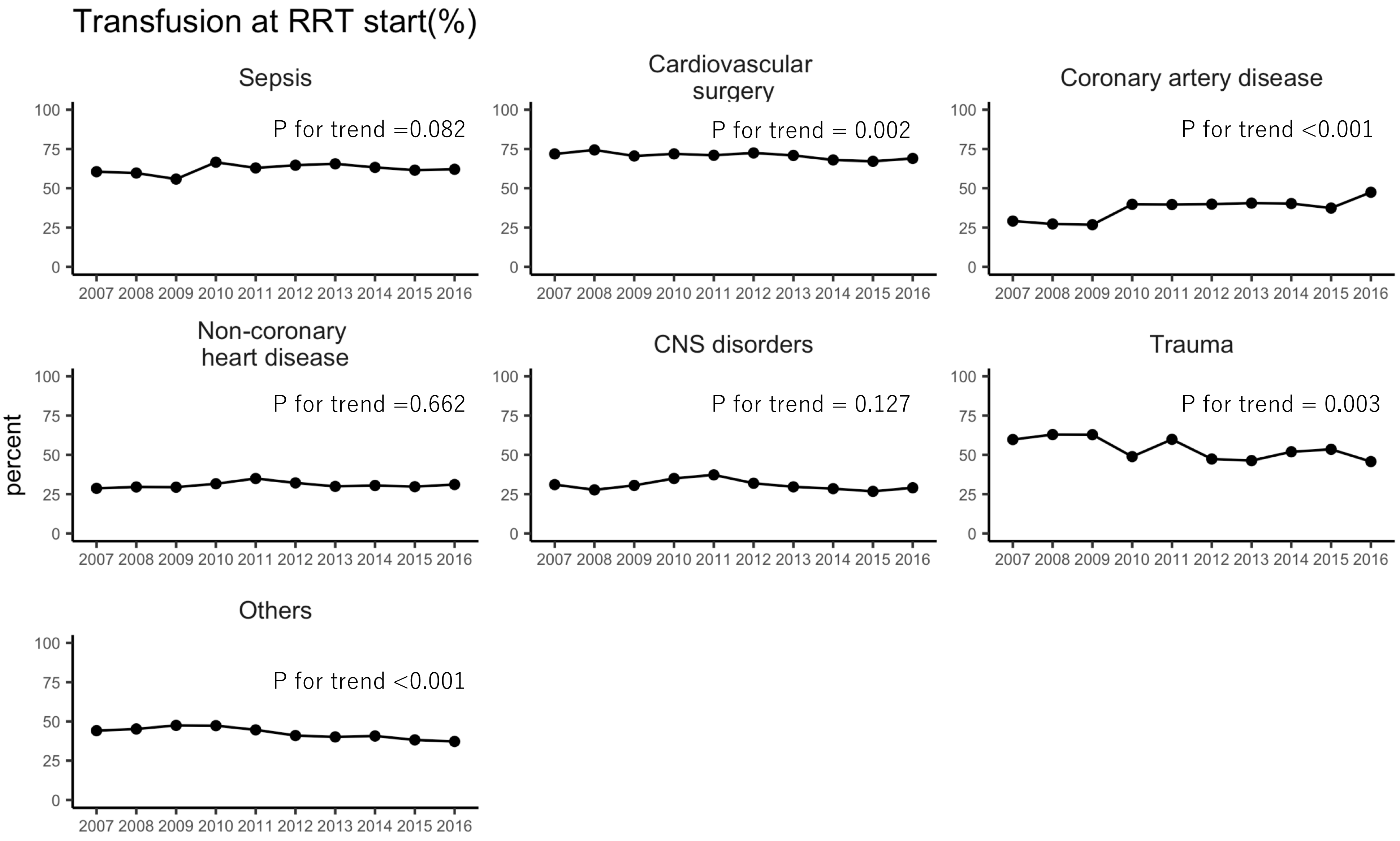

Transfusion at RRT start(%)
Sepsis
P for trend =0.082
Cardiovascular surgery
P for trend = 0.002
Coronary artery disease
P for trend <0.001
Non-coronary heart disease
P for trend =0.662
CNS disorders
P for trend = 0.127
Trauma
P for trend = 0.003
Others
P for trend <0.001
percent
0 25 50 75 100
2007 2008 2009 2010 2011 2012 2013 2014 2015 2016

File 4. Subgroup analyses: in-hospital mortality of patients treated with renal replacement therapy for acute kidney injury in intensive care units from 2007 to 2016

Year	2007	2008	2009	2010	2011	2012	2013	2014	2015	2016	P for trend
Subgroup by age group											
i) < 65	36.9	37.5	37.2	37.3	34.2	32.5	32.9	32.6	33.1	30.1	P<0.001
ii) 65-74	45.1	42.6	45.8	44.6	41.4	39.3	34.5	37.8	34.8	34.9	P<0.001
iii) 75-84	52.0	49.8	49.8	48.9	46.9	46.7	44.3	42.7	39.6	39.9	P<0.001
iv) > 85	54.2	60.3	55.9	51.8	55.3	50.5	49.4	50.1	48.4	46.9	P<0.001
Subgroup by sex											
i) Male	44.3	43.7	44.9	44.1	41.9	40.6	38.6	38.7	36.9	35.8	P<0.001
ii) Female	46.0	45.1	45.1	43.8	41.6	39.5	37.6	38.8	37.2	36.6	P<0.001
Subgroup by CKD											
i) With CKD	31.7	28.0	30.2	25.2	25.0	24.3	22.9	23.0	21.1	21.3	P<0.001
ii) Without CKD	49.3	49.9	49.9	50.4	47.8	46.1	43.8	44.5	42.7	41.5	P<0.001
Subgroup by mechanical ventilation											
i) Receiving mechanical ventilation at RRT start	60.0	57.8	58.0	58.5	55.1	53.8	50.5	52.7	51.0	50.3	P<0.001
ii) Not receiving mechanical ventilation at RRT start	29.9	30.1	30.7	27.7	27.8	26.0	26.0	24.9	23.1	21.3	P<0.001

File 5. The proportion of RRT dependence at discharge stratified by CKD, in patients treated with renal replacement therapy for acute kidney injury in intensive care units from 2007 to 2016

Year	2007	2008	2009	2010	2011	2012	2013	2014	2015	2016	P for trend
i) With CKD	37.6	40.4	39.9	43.5	44.6	47.1	46.9	47.8	47.5	46.5	P<0.001
ii) Without CKD	8.6	8.9	7.5	7.9	8.8	9.0	8.9	8.9	8.8	8.8	P<0.001
